# Brevianamide F Exerts Antithrombotic Effects by Modulating the MAPK Signaling Pathway and Coagulation Cascade

**DOI:** 10.3390/md22100439

**Published:** 2024-09-26

**Authors:** Huiwen Zhang, Chen Sun, Qing Xia, Peihai Li, Kechun Liu, Yun Zhang

**Affiliations:** 1 Biology Institute, Qilu University of Technology (Shandong Academy of Sciences), Jinan 250103, China; lyzhanghuiwen@163.com (H.Z.);; 2 Engineering Research Center of Zebrafish Models for Human Diseases and Drug Screening of Shandong Province, Jinan 250103, China

**Keywords:** Brevianamide F, marine natural products, antithrombotic, zebrafish, arachidonic acid

## Abstract

Existing antithrombotic drugs have side effects such as bleeding, and there is an urgent need to discover antithrombotic drugs with better efficacy and fewer side effects. In this study, a zebrafish thrombosis model was used to evaluate the antithrombotic activity and mechanism of Brevianamide F, a deep-sea natural product, with transcriptome sequencing analysis, RT-qPCR analysis, and molecular docking. The results revealed that Brevianamide F significantly attenuated the degree of platelet aggregation in the thrombus model zebrafish, leading to an increase in the number of circulating platelets, an augmentation in the return of blood to the heart, an elevated heart rate, and a significant restoration of caudal blood flow velocity. Transcriptome sequencing and RT-qPCR validation revealed that Brevianamide F may exert antithrombotic effects through the modulation of the MAPK signaling pathway and the coagulation cascade reaction. Molecular docking analysis further confirmed this result. This study provides a reference for the development of therapeutic drugs for thrombosis.

## 1. Introduction

Thrombotic disorder is a disease that occurs during thrombosis and thromboembolism pathology and is characterized by high morbidity and lethality [1]. The main causes include increased platelet counts, vessel wall damage, and decreased anticoagulant activity [2]. Depending on the mechanism of formation, thrombosis is mainly categorized into venous thrombosis and arterial thrombosis. Cardiovascular disease, the leading cause of death worldwide, accounts for a large proportion of arterial thrombotic disease. Venous thrombotic disease has a high incidence in hospitalized and post-operative patients and should not be ignored. It has been reported that one in four deaths worldwide is accompanied by thrombotic disorders, making antithrombotic drugs one of the hotspots in drug development [3,4,5]. Current antithrombotic drugs are limited by adverse effects, such as bleeding, thrombocytopenia, leukocytoclastic vasculitis, etc., as well as drug–drug interactions, to varying degrees [6,7,8]. Therefore, there is an urgent need to find new antithrombotic drugs.

The zebrafish model is characterized by its small size, transparent body, and thrombosis mechanism similar to that of humans. Zebrafish platelets are homologous to mammalian platelets, and their thrombosis mechanism is similar to that of humans, suggesting that zebrafish can be used as a model for screening antithrombotic drugs [9]. The green fluorescent labeling of platelet transgenic zebrafish *Tg(cd41:eGFP)* and the red fluorescent labeling of cardiac erythrocyte transgenic zebrafish *Tg(gata1a:dsRed)* can be used to visually monitor thrombus formation and development in real time. Furthermore, only a small amount of compounds are needed to evaluate antithrombotic activity using the zebrafish model, making it appropriate for the high-throughput screening of trace amounts of marine natural active products.

The unique environment of low temperature, high pressure, high salt, and low oxygen in the ocean has formed many natural products with novel structure and excellent pharmacological activity. For instance, marine biogenic heparin has fewer side effects and can lower the risk of bleeding in patients compared to land-based heparin sources [10,11]. It suggests that screening natural products from the ocean for antithrombotic activity is an important way to seek new antithrombotic drugs. At present, the activities of substances that have been isolated from deep-sea microorganisms are mostly focused on anti-tumor, anti-inflammatory, anti-viral, and anti-tuberculosis properties, while there is a relative lack of research on anti-thrombosis [12,13,14,15].

Brevianamide F is the parent nucleus of indole diketopiperazine analogs consisting of tryptophan and proline, a marine natural compound [16,17,18]. Recent studies on Brevianamide F have focused on the activities of its derivatives. For example, Brevianamide F analogues have bacteriostatic, anti-tumor, and insecticidal effects [19,20,21]. However, there are few studies on the activity of Brevianamide F. In particular, the antithrombotic activity of Brevianamide F has not been described.

In this study, a zebrafish thrombus model induced by arachidonic acid (AA) was used to evaluate the antithrombotic activity of deep-sea natural product Brevianamide F, with the degree of platelet aggregation, circulating platelet count, cardiac erythrocyte staining area and intensity, red blood cell aggregation rate in the middle part of the body, cardiac erythrocyte fluorescence area, heart rate, and caudal blood flow velocity as evaluation indexes. The mechanism of its antithrombotic action was explored using transcriptome sequencing, qRT-PCR assays, and molecular docking techniques.

## 2. Results

### 2.1. Effect of Brevianamide F on Platelet Aggregation and Circulating Platelet Count in Thrombotic Zebrafish

**Figure 1:**
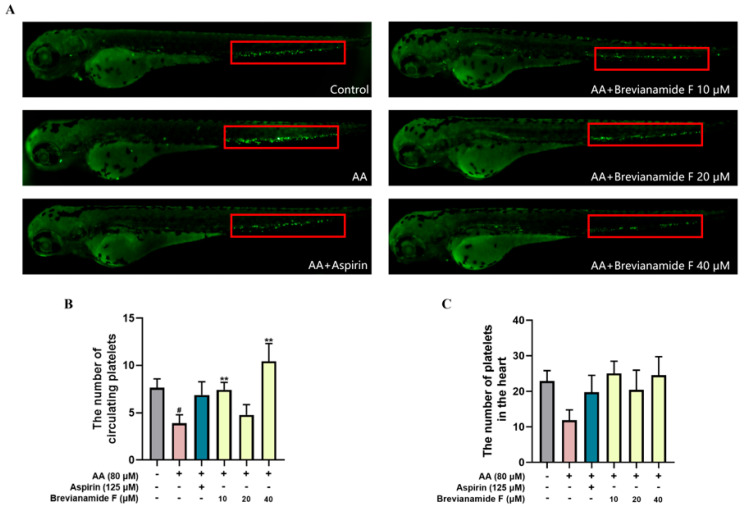
(**A**) Platelet distribution of zebrafish in each group, with tail platelet aggregation in the red frame. (**B**) The number of platelets circulating in the caudal. (**C**) The number of platelets in the heart. ^#^ *p* < 0.05, compared with the blank control group; ** *p* < 0.01, compared with the arachidonic acid (AA) group.

Arachidonic acid leads to thrombosis by causing platelet aggregation. The zebrafish in the thrombus model group showed platelet aggregation in their tails, and their circulating platelet count was lower than that of the blank control group. Platelet aggregation was reduced in the tails of zebrafish and the number of circulating platelets in the caudal was increased in the Aspirin treatment group and the Brevianamide-F-administered groups (10 μM, 20 μM, and 40 μM) compared with the thrombus model group (Figure 1A,B). Figure 1C demonstrates that there were fewer circulating platelets in the heart in the model group as opposed to the blank control group. The group that received Brevianamide F showed a tendency toward more circulating platelets in their hearts when compared to the model group, although there was no statistically significant difference.

### 2.2. Effect of Brevianamide F on the Staining Area and Intensity of Erythrocytes in the Heart of Thrombotic Zebrafish

**Figure 2:**
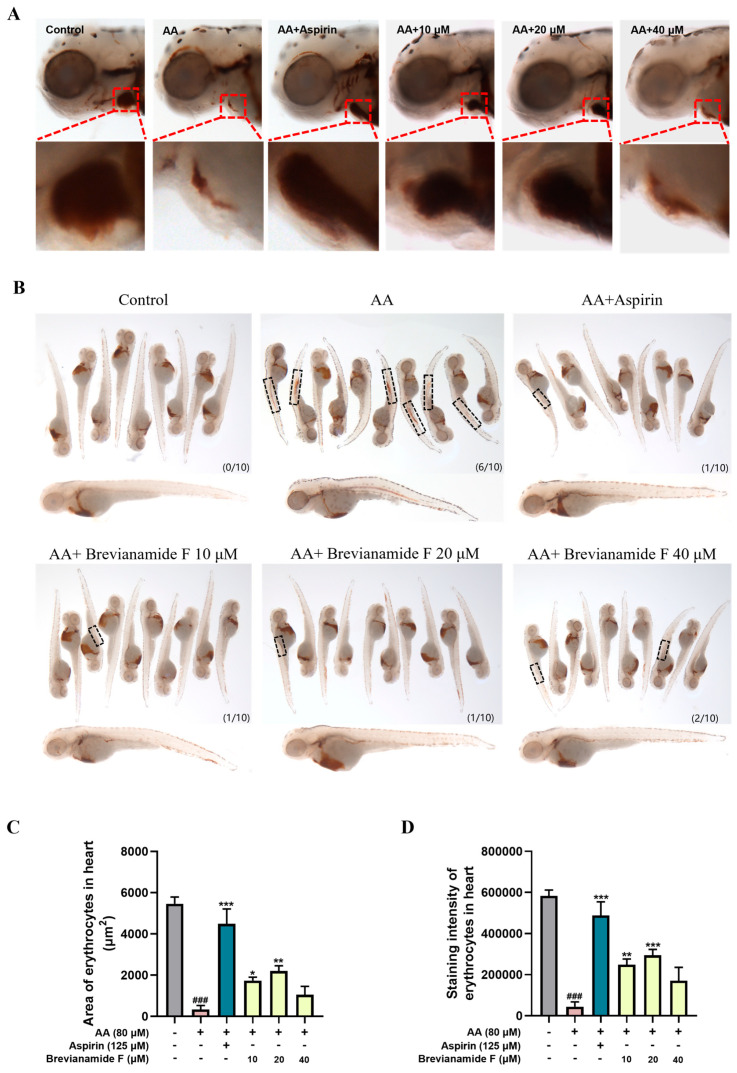
Representative images of heart region (**A**) and trunk region (**B**) of zebrafish stained with O-dianisidine in each group. The black frame indicates erythrocyte aggregation; the lower right corner was labeled as the number of zebrafish with erythrocyte staining in the trunk region/total number of zebrafish. Erythrocyte staining area (**C**) and staining intensity (**D**) in the heart. ^###^ *p* < 0.001, compared with the blank control group; * *p* < 0.05, ** *p* < 0.01, and *** *p* < 0.001, compared with the AA group.

O-dianisidine staining can reflect the distribution of erythrocytes. The normal zebrafish were stained by O-dianisidine with erythrocytes in the heart area and no erythrocyte aggregation in the trunk and tail. Compared with the blank control group, 60% of zebrafish in the thrombus model group appeared to exhibit erythrocyte aggregation in the middle part of the body, with a significant decrease in the number of cardiac erythrocytes and a significant decrease in staining intensity, indicating successful modeling (Figure 2A,B). Following treatment with the positive drug Aspirin and different concentrations of Brevianamide F, the percentage of erythrocyte aggregated in the trunk region of zebrafish was reduced, and the staining area and intensity of cardiac erythrocytes were significantly increased compared with those of the AA group (Figure 2C,D). Brevianamide F at a concentration of 20 μM had a 43.13% preventive effect on thrombosis (Table 1).

### 2.3. Effect of Brevianamide F on Erythrocyte Fluorescence Area and Heart Rate of Thrombotic Zebrafish

**Figure 3:**
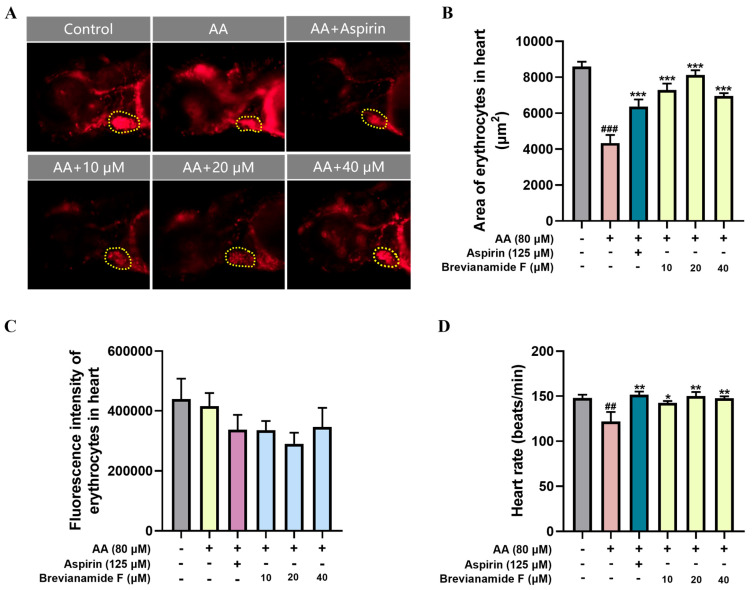
(**A**) Representative images of the heart region, (**B**) cardiac erythrocyte fluorescence area, (**C**) cardiac erythrocyte fluorescence intensity, and (**D**) heart rate. ^##^ *p* < 0.01 and ^###^ *p* < 0.001, compared with the blank control group; * *p* < 0.05, ** *p* < 0.01, and *** *p* < 0.001, compared with the AA group.

The transgenic zebrafish *Tg*(*gata1a:DsRed*) with red fluorescent protein-labeled cardiac erythrocytes showed the volume of blood that was returned to the heart in zebrafish. As depicted in Figure 3, the cardiac erythrocyte fluorescence area was significantly reduced and the heart rate was lower in the thrombus model group compared with the blank control group. The Brevianamide-F-treated groups (10 μM, 20 μM, and 40 μM) all showed a significant increase in the cardiac erythrocyte fluorescence area and a remarkable elevation in heart rate compared with the thrombus model group. However, there was no significant change in cardiac fluorescence intensity between groups.

### 2.4. Effect of Brevianamide F on Blood Flow Velocity in the Caudal Region of the Thrombotic Zebrafish

**Figure 4:**
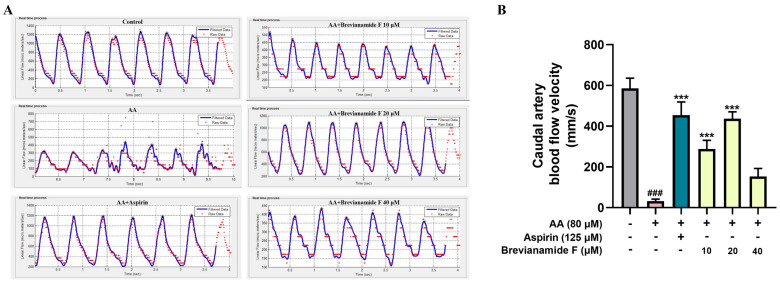
(**A**) Hemodynamic images of the caudal arteries of zebrafish in each group. (**B**) Histogram of blood flow velocity in the caudal artery. ^###^ *p* < 0.001, compared with the blank control group; *** *p* < 0.001, compared with the AA group.

The zebrafish with platelet aggregation thrombosis had slower blood flow velocities compared to normal zebrafish. Figure 4 reveals that the zebrafish in the thrombus model group had much lower caudal artery flow velocity than those in the blank control group. Brevianamide F exhibited noteworthy antithrombotic activity in comparison to the thrombus model group, as evidenced by a significant increase in the caudal artery blood flow velocity of zebrafish in the treatment groups.

### 2.5. Effect of Brevianamide F on Thrombosis-Related Factors

Thromboxane A2 (TXA_2_) is a cyclooxygenase metabolite of AA that strongly contracts blood vessels and promotes platelet aggregation [22]. von Willebrand Factor (vWF) is an important plasma constituent that connects collagen fibers and platelets to form thrombi [23]. Platelets adhere to exposed collagen and vWF, further releasing TXA_2_ and activating surrounding platelets to promote thrombus formation. D-dimer (D-dimer) is an important molecular marker of thrombus formation and the activation of the fibrinolytic system, which can reflect the severity of thrombus [24]. In this study, ELISA was used to determine the levels of TXA_2_, vWF, and D-dimer in zebrafish.

**Figure 5:**
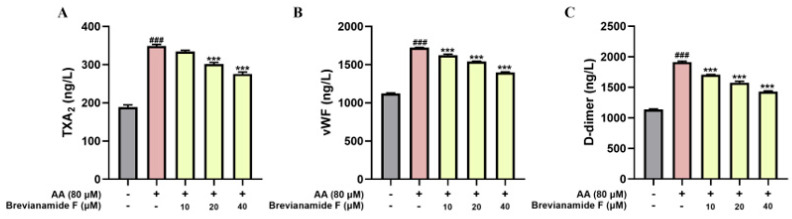
The levels of (**A**) TXA_2_, (**B**) vWF, and (**C**) D-dimer in zebrafish. ^###^ *p* < 0.001, compared with the blank control group; *** *p* < 0.001, compared with the AA group.

The contents of thrombus-associated factors TXA_2_, vWF, and D-dimer were significantly increased after AA treatment, and the contents of TXA_2_, vWF, and D-dimer were significantly decreased after the intervention of Brevianamide F at different concentrations, as illustrated in Figure 5A–C. The findings manifested that AA induced the expression of thrombus-related factors in zebrafish, and Brevianamide F inhibited AA-induced thrombus formation in zebrafish to varying degrees. This indicates that Brevianamide F could exert antithrombotic effects by suppressing the expression of thrombosis-related factors.

### 2.6. Effect of Brevianamide F on Gene Expression Profile of Thrombotic Zebrafish

#### 2.6.1. Differentially Expressed Genes (DEGs) Using Transcriptome Analysis

**Figure 6:**
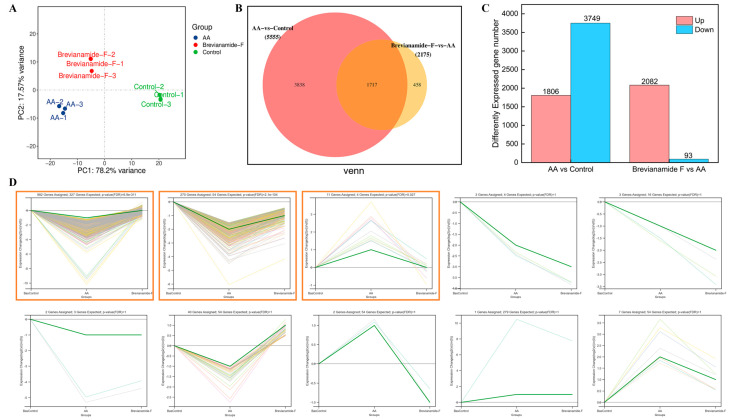
(**A**) Principal Component Analysis (PCA) plot, (**B**) Venn diagram of common and specific differentially expressed genes (DEGs) between different groups, (**C**) statistical histogram of DEGs, and (**D**) intersection DEG trend analysis. Orange boxes indicate the DEGs with significant change trends.

Transcriptome sequencing was performed to further investigate the mechanism of the antithrombotic action of Brevianamide F. Principal Component Analysis (PCA) plots showed compact sample clustering between groups, indicating low sample variability (Figure 6A). Genes with *p* values < 0.05 and |log_2_ (fold change)| >1 were classified as differentially expressed genes (DEGs). A total of 5555 DEGs, including 1806 up-regulated genes and 3749 down-regulated genes, were identified in the model group compared with the blank control group. A total of 2175 DEGs, including 2082 up-regulated genes and 93 down-regulated genes, were identified in the Brevianamide-F-treated group compared with the model group (Figure 6B,C). There were 1717 common DEGs between the above two comparison groups, and these genes were analyzed for trends. Among them, 1252 DEGs had low expression in the model group and high expression in the Brevianamide F group; 11 DEGs had high expression in the model group and low expression in the Brevianamide F group (Figure 6D).

#### 2.6.2. GO Function Enrichment Analysis and KEGG Enrichment Analysis

**Figure 7:**
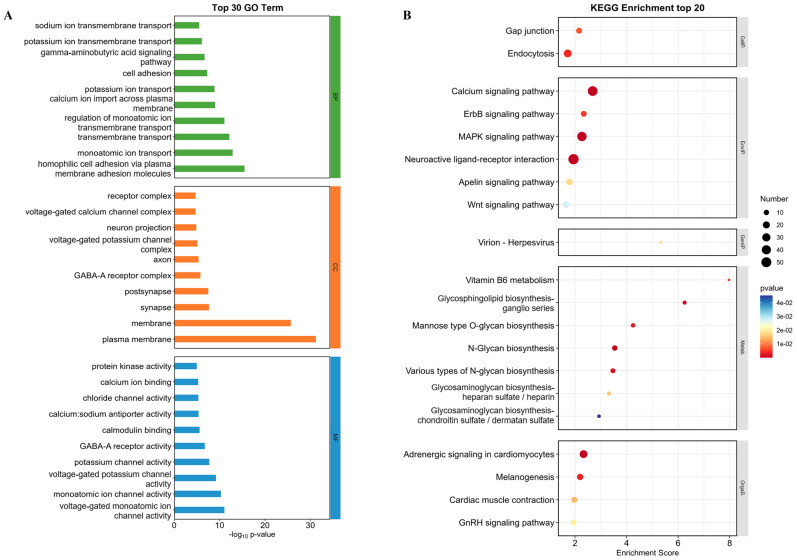
The effect of Brevianamide F on intersection DEGs with significant trend changes. (**A**) The GO function enrichment analysis. The vertical axis was a GO term which included three categories: biological process (BP), cellular component (CC), and molecular function (MF). The horizontal axis was the -log_10_(*p*-value) of the GO items. (**B**) The KEGG pathway enrichment analysis. The vertical axis was the name of the signaling pathway and the horizontal axis was the enrichment score. The size of the scatter represented the number of DEGs of the item, and the redder the color, the smaller the enrichment *p*-value and the greater the significance of the item.

GO function enrichment analysis and KEGG enrichment analysis were performed on DEGs with significant trends to explore the mechanism of Brevianamide F antithrombotic action. The 30 most significant GO enrichment terms were selected and displayed as histograms. The biological processes (BPs) significantly affected by Brevianamide F treatment were mainly related to cell adhesion (GO:0007156 and GO:0007155) and the regulation of ion transmembrane transport (GO:0006811, GO:0055085, GO:0034765, etc.). The cellular components (CCs) included plasma membranes (GO:0005886), synapse (GO:0045202), post-synapse (GO:0098794), and so on. The molecular function (MF) mainly included ion channel activity (GO:0005244, GO:0005216, GO:0005267, etc.) and protein kinase activity (GO:0004672) (Figure 7A).

The KEGG enrichment analysis showed that the DEGs were enriched in the MAPK signaling pathway, the calcium signaling pathway, and the apelin signaling pathway. The organismal systems and metabolic processes involved were as follows: adrenergic signaling in cardiomyoctes (dre04261), glycosaminoglycan biosynthesis in heparan sulfate and heparin (dre00534), and glycosaminoglycan biosynthesis in chondroitin sulfate and dermatan sulfate (dre00532) (Figure 7B). The above results demonstrated that Brevianamide F may exert antithrombotic effects by regulating signaling pathways (such as MAPK) and promoting the synthesis of antithrombotic active substances (such as heparin and chondroitin sulfate).

#### 2.6.3. Effect of Brevianamide F on the Gene Expression Levels of Zebrafish

**Figure 8:**
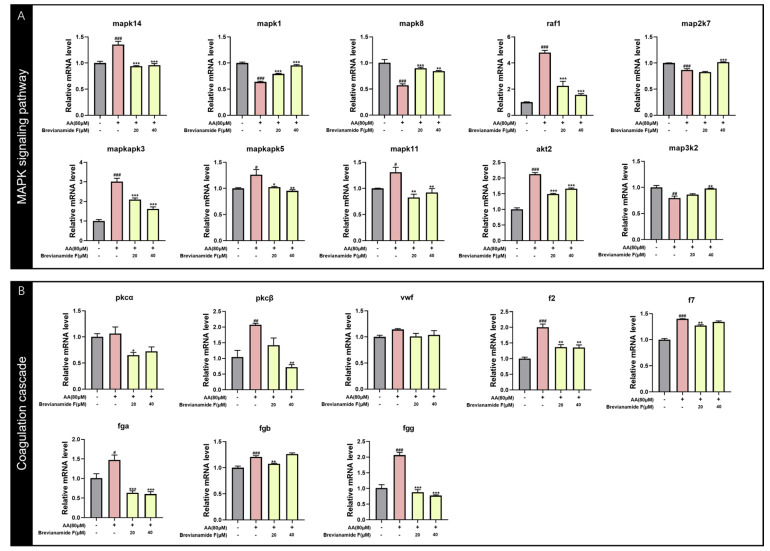
Effects of Brevianamide F on the expression of genes related to the MAPK pathway and coagulation cascade; ^#^ *p* < 0.05, ^##^ *p* < 0.01, and ^###^ *p* < 0.001, compared with the blank control group; * *p* < 0.05, ** *p* < 0.01, and *** *p* < 0.001, compared with the AA group.

Combined with the results of the trend analysis, as well as GO and KEGG enrichment analyses, the DEGs related to the MAPK pathway were selected for qRT-PCR. Figure 8A shows that the mRNA expression levels of MAPK-related genes *mapkapk3*, *mapkapk5*, *raf1*, *mapk11*, *mapk14*, and *akt2* were significantly up-regulated, and the expression levels of *map3k2*, *map2k7*, *mapk1*, and *mapk8* were significantly down-regulated in the model group compared with the blank control group. The levels of the above genes were notably regulated in the Brevianamide-F-treated group compared to the model group. The expression levels of genes related to the coagulation cascade response, which were closely related to thrombus formation and development, were also examined. AA induction elevated the expression levels of the thrombus-related factors *PKCα*, *PKCβ*, *vWF*, *f2*, *f7*, *fga*, *fgb*, and *fgg*. The expression levels of the above thrombosis-related factors were significantly decreased via Brevianamide F intervention (Figure 8B). These results showed that the mechanism of the antithrombotic effect of Brevianamide F may be related to the regulation of the MAPK signaling pathway, the inhibition of platelet activation, and the expression of mRNAs related to the coagulation cascade.

### 2.7. Interactions of Brevianamide F with Key Targets

**Figure 9:**
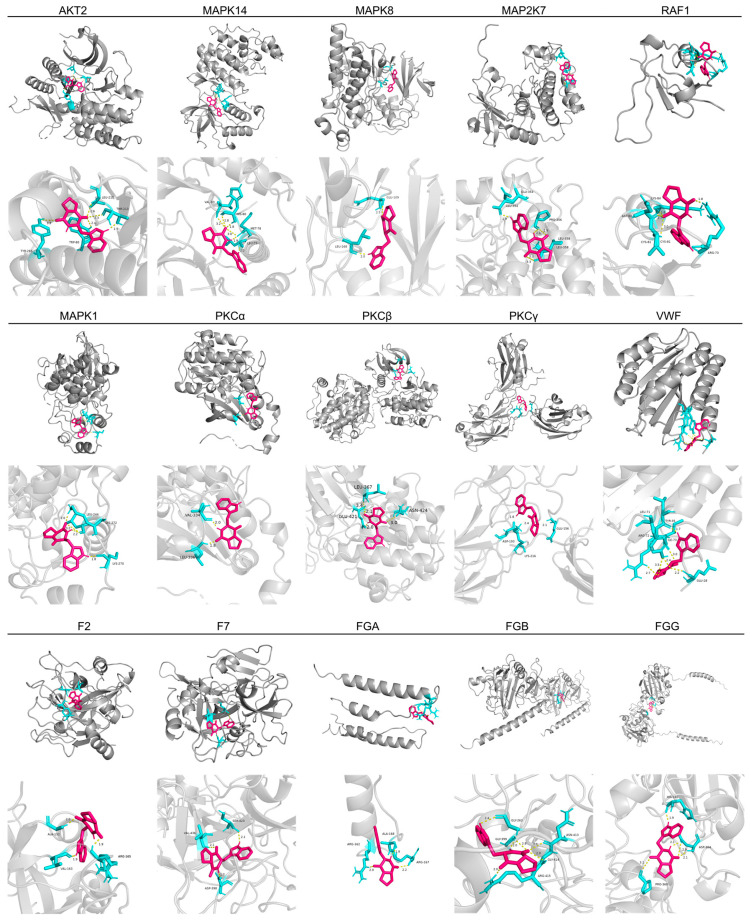
The molecular docking results of Brevianamide F with key targets. Each result contains an overall picture and a local zoomed-in picture. Red color indicates the molecular structure of Brevianamide F. Hydrogen bonds are shown in yellow and amino acid residues are shown in blue.

To further corroborate the mechanism of the antithrombotic action of Brevianamide F, molecular docking was used to assess the potential interaction of Brevianamide F with key targets. Combined with the literature and qRT-PCR assay results, key targets in the MAPK signaling pathway and coagulation cascades AKT2, MAPK14, MAPK8, MAP2K7, RAF1, MAPK1, PKCα, PKCβ, PKCγ, VWF, F2, F7, FGA, FGB, and FGG were selected and docked with Brevianamide F using AutoDock software (Table 2). The binding energies less than 0 kJ/mol indicated that Brevianamide F was capable of spontaneously binding to key target proteins. Meanwhile, less than −5 kJ/mol indicated stable binding [25,26,27]. Conformation with the lowest binding energy was chosen as the final docking result of Brevianamide F in relation to the key targets and the results were then visualized using PyMOL software (Figure 9). The molecular docking results revealed that the binding energies of Brevianamide F to the key targets of the MAPK/coagulation cascade were lower than −5 kJ/mol. Among the calculated binding energies, the binding energies of Brevianamide F to F7, MAPK14, MAP2K7, and AKT2 were −40.75 kJ/mol, −36.94 kJ/mol, −34.81 kJ/mol, and −34.43 kJ/mol, respectively, suggesting that F7, MAPK14, MAP2K7, and AKT2 may be important targets for the antithrombotic effects of Brevianamide F.

## 3. Discussion

Arachidonic-acid-induced thrombosis is a commonly used way of modeling zebrafish thrombosis and is based on the principle of causing platelet aggregation and regulating vasoconstriction (resulting in thrombosis) [28,29,30]. It has been reported that the staining of erythrocytes in the heart and the accumulation of erythrocyte aggregation in the tails of zebrafish can reflect the degree of thrombosis [31]. When thrombus occurred, the staining area and intensity of erythrocyte decreased in the heart but increased in the tail. In this experiment, the thrombus model group showed an increase in tail platelet aggregation, a decrease in the number of circulating platelets, and a decrease in the staining area and intensity of cardiac erythrocyte compared with the blank control group. At the same time, 60% of the zebrafish showed erythrocyte aggregation in the middle of the body, indicating that the modeling was successful. Aspirin was a well-recognized antiplatelet aggregating agent and was widely used in antithrombotic therapy. It blocked thrombus formation by inhibiting cyclooxygenase and preventing AA-induced platelet aggregation [8]. Therefore, this study set up the thrombosis model group, the Aspirin-positive control group, and the different concentration Brevianamide F treatment group. The results suggested that Brevianamide F exhibited significant antithrombotic effects. Compared with the AA group, Brevianamide F significantly reduced platelet aggregation, increased the return blood volume in heart, elevated the heart rate, and restored the blood flow velocity in the caudal artery of zebrafish.

Thromboxane A2 (TXA_2_) was formed by the action of related enzymes when AA activated platelets. TXA_2_ promoted thrombosis by facilitating platelet aggregation and vasoconstriction [8]. The vWF was an important plasma component that bound platelets and collagen fibers to form thrombus, thereby exerting a hemostatic effect [23]. D-dimer was produced after the degradation of cross-linked fibrin, suggesting that intravascular coagulation can be used as a marker of coagulation and fibrinolytic system activation [32]. In this study, the levels of TXA_2_, vWF, and D-dimer in zebrafish were determined using the ELISA assay. The results showed that Brevianamide F exerted antithrombotic effects by reducing the levels of TXA_2_, vWF, and D-dimer. To further elucidate the mechanism of the antithrombotic action of Brevianamide F, transcriptome sequencing, the trend analysis of differentially expressed genes, GO, and KEGG enrichment analyses were performed. The results demonstrated that the antithrombotic mechanism of Brevianamide F was related to the MAPK signaling pathway and the coagulation cascade response.

The MAPK signaling pathway played a key role in the pathogenesis of several cardiovascular diseases. It has been found that p38 MAPK, ERK1/2, and JNK1/2 were expressed in platelets and were important regulators of platelet activation [33,34]. Raf1, a serine/threonine kinase, was required for MEK1/2 phosphorylation. It was involved in the regulation of vascular endothelial cell proliferation and angiogenesis. These processes are closely related to thrombosis [35,36]. MAP2K7 was an upstream activator of JNK, which affected thrombosis by regulating the JNK signaling pathway [37]. Previous studies have shown that AA significantly elevated the expression of genes related to the MAPK signaling pathway, such as *p38 mapk (mapk14)*, *mapk1 (erk)*, *mapk8 (jnk)*, and *raf1*, and increased the phosphorylation of key proteins of the pathway (e.g., p38 MAPK, ERK1/2, and JNK1/2) [35,38,39]. Akt2 was a vital factor in the process of thrombosis. It has been shown that platelets from Akt2-/- mice were significantly defective in aggregation and fibrinogen binding [40]. Yin et al. found that akt2 regulated the VWF/GPIb-IX-induced signaling pathway, leading to platelet activation, which in turn promoted platelet adhesion, diffusion, and aggregation [41]. In this study, the expression levels of genes (*mapk14*, *raf1*, *mapkapk3*, *mapkapk5*, *mapk11*, and *akt2*) related to the MAPK signaling pathway were significantly up-regulated by AA. Treatment with Brevianamide F reversed the trend of these genes. In addition, some researches had also found that the thrombus model reduced the phosphorylation of JNK, and the administration of paeoniflorin with antithrombotic activity significantly increased the phosphorylation of JNK [42]. This might be caused by the negative feedback regulation of the organisms. In this study, AA significantly down-regulated *mapk1*, *mapk8*, *map2k7*, and *map3k2*. Treatment with Brevianamide F rescued the aberrant expression of these genes. The results above demonstrated that Brevianamide F could effectively prevent thrombus formation and maintain cellular homeostasis.

In the process of coagulation, the MAPK signaling pathway may promote the coagulation cascade by affecting platelet activation, endothelial cell function, and the expression and activity of coagulation factors [43,44]. The factors associated with platelet activation and the coagulation cascade hugely affected the formation and development of thrombus. The PKCα and PKCβ were two different isoforms of the protein kinase C (PKC) family, which were predominantly expressed in platelets. They affected thrombosis by activating platelet activation, aggregation, and clotting factor release [45,46]. The vWF was an important plasma protein that played a key role in blood coagulation. It promoted platelet adhesion at the site of vascular injury to achieve hemostasis [31,47,48]. Coagulation factor Ⅶ (f7) was a vital factor in the external coagulation pathway and could form a complex with tissue factors to initiate the formation of fibrin. The FGA (fibrinogen A chain), FGB (fibrinogen B chain), and FGG (fibrinogen gamma chain) were the three subunits of fibrinogen. Fibrinogen was converted to fibrin during blood clotting and promoted the stability of blood clots [49,50]. Previous studies have shown that the gene expression of thrombosis-related factors (such as *pkcα*, *pkcβ*, *vwf*, *f2*, *f7*, *fga*, *fgb*, and *fgg*) can promote platelet activation, increase fibrinogen synthesis, enhance platelet aggregation, and induce thrombosis [51,52]. In this study, AA significantly increased the mRNA expression of *pkcα*, *pkcβ*, *vwf*, *f2*, *f7*, *fga*, *fgb*, and *fgg*, whereas the intervention of Brevianamide F significantly inhibited the mRNA expression of these factors, suggesting that Brevianamide F exerted antithrombotic effects through the inhibition of platelet activation and the coagulation cascade.

Molecular docking was a useful tool for discovering active compounds and exploring their mechanisms of action, providing new scientific and technological support for the research and development of marine active compounds [53]. In order to further elucidate the molecular mechanism by which Brevianamide F exerted antithrombotic activity, molecular docking between Brevianamide F and key target proteins related to the MAPK signaling pathway and the coagulation cascade was carried out. It was found that Brevianamide F bound stably to the key targets, such as F7, MAPK14, MAP2K7, and AKT2.

The results above suggested that Brevianamide F exerted antithrombotic effects by regulating the MAPK signaling pathway and inhibiting the platelet activation and coagulation cascade. This study provided a reference for the discovery of Marine antithrombotic drugs.

## 4. Materials and Methods

### 4.1. Chemicals and Reagents

Brevianamide F and O-dianisidine were purchased from Shanghai Aladdin Biochemical Technology Co., Ltd. (Shanghai, China). Arachidonic acid was purchased from Stanford Analytical Chemicals Inc. (Denver, CO, USA). Aspirin was purchased from Beijing Puxi Standard Technology Co., Ltd. (Beijing, China). Phenylthiourea was obtained from Sigma-Aldrich (Shanghai, China) Trading Co., Ltd. (Shanghai, China). The experimental water was used for zebrafish culture (5.0 mM NaCl, 0.17 mM KCl, 0.4 mM CaCl_2_, and 0.16 mM MgSO_4_).

### 4.2. Zebrafish Maintenance

The wild-type AB strain zebrafish, transgenic zebrafish *Tg(cd41:eGFP)* with green fluorescent protein-labeled platelets, and *Tg(gata1a:DsRed)* with red fluorescent protein-labeled cardiac erythrocytes, supplied by the Engineering Research Center of Zebrafish Models for Human Diseases and Drug Screening of Shandong Province, were used in this study. Male and female zebrafish were kept separately under standard conditions of 14 h of illumination/10 h of darkness at 28 ± 0.5 °C, and they were fed with granular bait at regular intervals. Healthy and sexually mature zebrafish were taken and placed in the mating tank in the ratio of 2:2 or 1:2 for males and females. The embryos were washed three times with zebrafish embryo culture water, and then sterilized with 0.1% methylene blue solution and transferred into zebrafish embryo culture water at 28 ± 0.5 °C with light control. Phenylthiourea (PTU) was added at 6 hpf (hours post fertilization) to a final concentration of 0.03 mg/mL to inhibit melanogenesis and facilitate observation [54].

### 4.3. Chemical Treatment

Zebrafish larvae with normal development were selected at 72 hpf under the microscope and transferred into 24-well plates with 10 larvae per well. A blank control group (zebrafish culture water), a thrombus model group (80 μM AA), a positive control group (80 μM AA + 125 μM Aspirin), and a Brevianamide F treatment group (80 μM AA + 10 μM, 20 μM or 40 μM Brevianamide F) were set up for the experiment. The concentrations of AA and Aspirin were set according to the standards in published articles [39,55]. Every group was maintained at 28 ± 0.5 °C in an incubator with a constant temperature. After 6 h, the solution was sucked out, the blank control group was added with zebrafish culture water, and the other groups were replaced with AA solution with a final concentration of 80 μM and treated for 1.5 h in the dark. The experiment was conducted in triplicate.

### 4.4. Detection of Circulating Platelet Count

Zebrafish of the *Tg(cd41:eGFP)* strain were selected, and the experimental groups and drug treatments were the same as those discussed in Section 4.3. After treatment, 10 zebrafish larvae were randomly selected from each group, and the number of circulating zebrafish platelets within 15 s was recorded under an Olympus inverted fluorescence microscope (Olympus, Tokyo, Japan) and analyzed statistically.

### 4.5. Erythrocyte Staining Area and Intensity Detection

Wild-type AB strain zebrafish were used, and the experimental groups and drug treatments are shown in Section 4.3. The zebrafish were stained using 1.0 mg/mL of O-dianisidine staining solution for 10 min after the drug treatment. Ten zebrafish were randomly selected from each group, and the images were captured under an AXIO-V16 fluorescence microscope (Zeiss, Oberkochen, Germany), and the cardiac erythrocyte staining area and intensity (S) were measured and calculated using Image-Pro Plus 6.0 software. The aggregation of erythrocytes in the middle part of the body was counted to calculate the rate of thrombosis prevention. The thrombosis prevention rate (%) = (S _treatment_−S _model_)/(S _control_−S _model_) × 100%.

### 4.6. Cardiac Erythrocyte Fluorescence Area and Heart Rate Detection

The *Tg*(*gata1a:dsRed*) strain of zebrafish was used, and the experimental groups and drug treatments are shown in Section 4.3. Ten zebrafish were randomly selected from each group, the images of the zebrafish cardiac region were captured, and heart rate was recorded under a Zeiss fluorescence microscope (Zeiss, Oberkochen, Germany). The area of erythrocyte fluorescence of the zebrafish in the heart was measured and calculated via Image-Pro Plus 6.0 software.

### 4.7. Caudal Blood Flow Velocity Detection

Wild-type AB strain zebrafish were selected, and the experimental groups and drug treatments were the same as those outlined in Section 4.3. After the treatment, 10 zebrafish larvae were randomly selected from each group, and the blood flow in the caudal artery of zebrafish was recorded using the blood flow system Zebralab (ViewPoint, Lyon, France) for 10 s. Caudal blood flow velocity analysis was performed using MicroZebraLab BloodFlow 3.4.6 software (ViewPoint, Lyon, France).

### 4.8. Detection of Thrombosis-Related Factors

The zebrafish were grouped and treated with drug interventions, as described in Section 4.3. The zebrafish were collected following drug administration and washed three times with PBS. Tissue homogenates were obtained by crushing the tissue with a multi-sample cryo-mill. The concentrations of thromboxane A2 (TXA_2_), von Willebrand factor (vWF), and D-dimer, which are closely related to thrombus, were detected using ELISA kits according to the manufacturer’s instructions (Jiangsu Meimian industrial Co., Ltd., Jiangsu, China). Sixty zebrafish were used for each sample, and each experiment was performed three times.

### 4.9. Identification of Differentially Expressed Genes in Zebrafish Using RNA-Seq

The zebrafish in the thrombus model group, blank control group, and Brevianamide F (20 µM) group were collected after three enzyme-free water washes following drug administration. RNA-Seq analysis was carried out with the assistance of Ouyi Biotechnology Co., Ltd. (Shanghai, China). DESeq2 software version 3.19 was used to analyze the differentially expressed genes (DEGs). Genes with *p* values < 0.05 and |log_2_ (fold change)| >1 were classified as DEGs. Subsequently, the differentially expressed genes were subjected to trend analysis, gene ontology (GO) functional enrichment, and enrichment analyses of the Kyoto Encyclopedia of Genes and Genomes (KEGG) pathway to identify the functions and potential expression trends of the DEGs.

### 4.10. qRT-PCR Assay for Expression Levels of Thrombus-Related Genes

The samples of zebrafish in the blank control group, the thrombus model group, and the Brevianamide-F-treated groups (10 µM, 20 µM, and 40 µM) were washed with purified water and then collected. Each group was conducted in triplicate. Total RNA was extracted using the FastPure Cell/Tissue Total RNA Isolation Kit V2 (Novozan, China) and reversed into cDNA using HiScript Ⅲ RT SuperMix for qPCR (+gDNA wiper) (Novozan, China). ChamQ Universal SYBR qPCR Master Mix (Novozan, China) was used to perform qRT-PCR on a LightCycler^®^96 (Roche, Switzerland). The running program was as follows: 30 s at 95 °C, followed by 45 cycles of 95 °C for 20 s and 72 °C for 30 s. rpl13a was used as a reference gene. The primer sequences for the genes used in this paper are shown in Table S1.

### 4.11. Molecular Docking

The molecular docking program AutoDock (4.2.6) was used to examine possible interactions of Brevianamide F with key targets associated with the coagulation cascade and MAPK signaling pathway. The structure of Brevianamide F was downloaded from PubChem (https://pubchem.ncbi.nlm.nih.gov/ accessed on 1 September 2024) and the binding energy was optimized by the MM2 algorithm using Chem3D software (20.0). The crystal structures of the key target proteins were directly obtained from the RCSB protein database (https://www.rcsb.org/ accessed on 1 September 2024). The related protein and Brevianamide F structures were processed using PyMOL software (2.6) and AutoDockTools-1.5.7 software. Docking was conducted by semi-flexible docking and was simulated 50 times. The lower the binding energy, the more stable the binding between Brevianamide F and the target protein.

### 4.12. Statistical Analysis

Statistical analysis was performed using Graphpad Prism 8.3.0 software, and experimental data were expressed as the mean ± standard error of the mean (mean ± SEM). The significant differences between groups were analyzed using one-way ANOVA (one-way ANOVA), with *p* < 0.05 indicating that the differences were statistically significant.

## 5. Conclusions

The deep-sea natural compound Brevianamide F possesses significant antithrombotic activity, which significantly increases the circulating platelet count, improves the return cardiac blood volume, decreases the erythrocyte aggregation rate in the middle part of the zebrafish body, restores the heart rate, and enhances the caudal blood flow velocity in the zebrafish thrombosis model. The mechanism of action of its antithrombotic activity may be related to the modulation of the MAPK signaling pathway and the coagulation cascade response. Therefore, Brevianamide F may be a promising candidate for the treatment of thrombotic diseases.

## Figures and Tables

**Table 1:** Preventive effects of each group on thrombosis in zebrafish.

Groups	Concentration (μM)	Cardiac Erythrocyte Staining Intensity (Pixel)	Thrombosis Prevention Rate (%)
Blank control group	–	583494 ± 28378	–
Thrombus model group (AA)	80	44318 ± 23093 ^###^	–
Positive control group (AA + Aspirin)	125	488484 ± 66153 ***	76.69
Brevianamide F treatment group (AA + Brevianamide F)	10	249285 ± 27242 *	35.39
	20	294097 ± 29205 **	43.13
	40	171199 ± 64751	21.91

^###^ *p* < 0.001, compared with the blank control group; * *p* < 0.05, ** *p* < 0.01, and *** *p* < 0.001, compared with the AA group.

**Table 2:** The binding energy of Brevianamide F with key targets.

Protein Target	PDB ID	Binding Energy (kJ/mol)
		Brevianamide F
AKT2	8Q61	−34.43
MAPK14	6SFI	−36.94
MAPK8	4QTD	−31.80
MAP2K7	5Y90	−34.81
RAF1	6VJJ	−32.38
MAPK1	6SLG	−28.79
PKCα	8U37	−30.08
PKCβ	2I0E	−29.75
PKCγ	2UZP	−31.00
VWF	7EOW	−33.93
F2	2AFQ	−32.13
F7	5PAG	−40.75
FGA	3E1I	−25.19
FGB	2OYH	−28.79
FGG	3E1I	−29.12

## Data Availability

The data presented in the current study are available on request from the corresponding author.

## Supplementary Materials

The following supporting information can be downloaded at: https://www.mdpi.com/article/10.3390/md22100439/s1. Table S1. The gene primers for Quantitative Real-time PCR.

## References

[1] Freund Y., Cohen-Aubart F., Bloom B. (2022). Acute pulmonary embolism: A review. JAMA.

[2] Buxhofer-Ausch V., Wolf D., Sormann S., Forjan E., Schimetta W., Gisslinger B., Heibl S., Krauth M.T., Thiele J., Ruckser R. (2021). Impact of Platelets on Major Thrombosis in Patients with a Normal White Blood Cell Count in Essential Thrombocythemia. Eur. J. Haematol..

[3] Raskob G.E., Angchaisuksiri P., Blanco A.N., Buller H., Gallus A., Hunt B.J., Hylek E.M., Kakkar A., Konstantinides S.V., McCumber M. (2014). Thrombosis. Arterioscl. Throm. Vas..

[4] Wendelboe A., Weitz J.-I. (2024). Global Health Burden of Venous Thromboembolism. Arterioscl. Throm. Vas..

[5] Roth G.A., Forouzanfar M.H., Moran A.E., Barber R., Nguyen G., Feigin V.L., Naghavi M., Mensah G.A., Murray C.J.L. (2015). Demographic and Epidemiologic Drivers of Global Cardiovascular Mortality. N. Engl. J. Med..

[6] Huang H.-K., Liu P.P.-S., Lin S.-M., Yeh J.-I., Hsu J.-Y., Peng C.C.-H., Munir K.M., Loh C.-H., Tu Y.-K. (2023). Risk of Serious Hypoglycemia in Patients with Atrial Fibrillation and Diabetes Concurrently Taking Antidiabetic Drugs and Oral Anticoagulants: A Nationwide Cohort Study. Eur. Heart J. Card. Pha..

[7] Lee J.Y., Oh I.-Y., Lee J.-H., Kim S.-Y., Kwon S.S., Yang H.-J., Kim Y.-K., Bang S.-M. (2020). The Increased Risk of Bleeding Due to Drug-Drug Interactions in Patients Administered Direct Oral Anticoagulants. Thromb. Res..

[8] Swan D., Loughran N., Makris M., Thachil J. (2020). Management of Bleeding and Procedures in Patients on Antiplatelet Therapy. Blood Rev..

[9] Jagadeeswaran P., Sheehan J.P., Craig F.E., Troyer D. (1999). Identification and Characterization of Zebrafish Thrombocytes. Brit. J. Haematol..

[10] Chandika P., Tennakoon P., Kim T.-H., Kim S.-C., Je J.-Y., Kim J.-I., Lee B., Ryu B., Kang H., Kim H.-W. (2022). Marine Biological Macromolecules and Chemically Modified Macromolecules; Potential Anticoagulants. Mar. Drugs.

[11] Chen J., Wang Z., Jia X., Li R., Chen J., Liu X., Song B., Zhong S., Qi Y. (2022). Anticoagulant and Fibrinolytic Properties of Two Heparinoid Compounds Prepared from Shrimp Waste. Foods.

[12] Wei J., Liu R., Hu X., Liang T., Zhou Z., Huang Z. (2021). MAPK Signaling Pathway-Targeted Marine Compounds in Cancer Therapy. J. Cancer Res. Clin..

[13] Linghu K.-G., Zhang T., Zhang G.-T., Lv P., Zhang W.-J., Zhao G.-D., Xiong S.-H., Ma Q.-S., Zhao M.-M., Chen M. (2024). Small Molecule Deoxynyboquinone Triggers Alkylation and Ubiquitination of Keap1 at Cys489 on Kelch Domain for Nrf2 Activation and Inflammatory Therapy. J. Pharm. Anal..

[14] Zhang K., Liang J., Zhang B., Huang L., Yu J., Xiao X., He Z., Tao H., Yuan J. (2024). A Marine Natural Product, Harzianopyridone, as an Anti-ZIKV Agent by Targeting RNA-Dependent RNA Polymerase. Molecules.

[15] Sun C., Liu Z., Zhu X., Fan Z., Huang X., Wu Q., Zheng X., Qin X., Zhang T., Zhang H. (2020). Antitubercular Ilamycins from Marine-Derived Streptomyces Atratus SCSIO ZH16 ΔilaR. J. Nat. Prod..

[16] Shi J., Wang C., Xie M., Hao Y., Wang N., Ma H., Yang X. (2022). Brefeldin A from the Deep-Sea-Derived Fungus Fusarium Sp. Targets on RIPK3 to Inhibit TNFα-Induced Necroptosis. Chem. Biodivers..

[17] Huang P., Xie F., Ren B., Wang Q., Wang J., Wang Q., Abdel-Mageed W.M., Liu M., Han J., Oyeleye A. (2016). Anti-MRSA and Anti-TB Metabolites from Marine-Derived Verrucosispora Sp. MS100047. Appl. Microbiol. Biot..

[18] Zhuravleva O.I., Afiyatullov S.S., Vishchuk O.S., Denisenko V.A., Slinkina N.N., Smetanina O.F. (2012). Decumbenone C, a New Cytotoxic Decaline Derivative from the Marine Fungus Aspergillus Sulphureus KMM 4640. Arch. Pharm. Res..

[19] Preciado S., Mendive-Tapia L., Torres-García C., Zamudio-Vázquez R., Soto-Cerrato V., Pérez-Tomás R., Albericio F., Nicolás E., Lavilla R. (2013). Synthesis and Biological Evaluation of a Post-Synthetically Modified Trp-Based Diketopiperazine. MedChemComm.

[20] Wauters I., Goossens H., Delbeke E., Muylaert K., Roman B.I., Van Hecke K., Van Speybroeck V., Stevens C.V. (2015). Beyond the Diketopiperazine Family with Alternatively Bridged Brevianamide F Analogues. J. Org. Chem..

[21] Li L., Chang Q.-H., Zhang S.-S., Yang K., Chen F.-L., Zhu H.-J., Cao F., Liu Y.-F. (2022). (±)-Brevianamides Z and Z1, New Diketopiperazine Alkaloids from the Marine-Derived Fungus Aspergillus Versicolor. J. Mol. Struct..

[22] Patel P., Naik M.U., Naik U. (2016). Apoptosis Signal-Regulating Kinase (ASK1) Regulates Thrombosis in Part By Regulating cPLA2 Phosphorylation-Dependent TxA2 Generation. Blood.

[23] Zhu S., Gilbert J.C., Hatala P., Harvey W., Liang Z., Gao S., Kang D., Jilma B. (2020). The Development and Characterization of a Long Acting Anti-thrombotic von Willebrand Factor (VWF) Aptamer. J. Thromb. Haemost..

[24] Gotta J., Gruenewald L.D., Geyer T., Eichler K., Martin S.-S., Mahmoudi S., Booz C., Biciusca T., Reschke P., Juergens L.-J. (2024). Indicators for Hospitalization in Acute Pulmonary Embolism: Uncover the Association Between D-dimer Levels, Thrombus Volume and Radiomics. Acad. Radiol..

[25] Wang D.-D., Yang Y., Hengerjia G., Deng Y. (2023). Exploring the mechanism of Liuwei Dihuang formula for promoting melanin synthesis in juvenile zebrafish based on network pharmacology and molecular docking. Heliyon.

[26] Gao Y., Guo Z., Liu Y. (2022). Analysis of the Potential Molecular Biology of Triptolide in the Treatment of Diabetic Nephropathy: A Narrative Review. Medicine.

[27] Qiu Y., Ying J., Yan F., Yu H., Zhao Y., Li H., Xia S., Chen J., Zhu J. (2023). Novel Antiosteoporotic Peptides Purified from Protein Hydrolysates of Taihe Black-Boned Silky Fowl: By Larval Zebrafish Model and Molecular Docking. Food Res. Int..

[28] Badimon L., Vilahur G., Rocca B., Patrono C. (2021). The Key Contribution of Platelet and Vascular Arachidonic Acid Metabolism to the Pathophysiology of Atherothrombosis. Cardiovasc. Res..

[29] Mi Y., Han X., Yu X., Li L., Tang X., Li G. (2024). Sarcocinerenolides A, an open-loop decarbonizing cembranolide, and sarcocinerenolides B–I, eight polyoxygenated cembranolides with anti-thrombotic activity from the South China Sea soft coral Sarcophyton cinereum. Phytochemistry.

[30] Wang Z., Liu P., Hu M., Lu S., Lyu Z., Kou Y., Sun Y., Zhao X., Liu F., Tian J. (2021). Naoxintong Restores Ischemia Injury and Inhibits Thrombosis via COX2-VEGF/NFκB Signaling. J. Ethnopharmacol..

[31] Yin S.-J., Luo Y.-Q., Zhao C.-P., Chen H., Zhong Z.-F., Wang S., Wang Y.-T., Yang F.-Q. (2020). Antithrombotic Effect and Action Mechanism of Salvia Miltiorrhiza and Panax Notoginseng Herbal Pair on the Zebrafish. Chin. Med..

[32] García Suquia A., Alonso-Fernández A., de la Peña M., Romero D., Piérola J., Carrera M., Barceló A., Soriano J.B., Arque M., Fernández-Capitán C. (2015). High D-Dimer Levels after Stopping Anticoagulants in Pulmonary Embolism with Sleep Apnoea. Eur. Resp. J..

[33] Misasi R., Capozzi A., Riitano G., Recalchi S., Manganelli V., Mattei V., Longo A., De Michele M., Garofalo T., Pulcinelli F.M. (2021). Signal Transduction Pathway Involved in Platelet Activation in Immune Thrombotic Thrombocytopenia after COVID-19 Vaccination. Haematologica.

[34] Fan X., Wang C., Shi P., Gao W., Gu J., Geng Y., Yang W., Wu N., Wang Y., Xu Y. (2018). Platelet MEKK3 Regulates Arterial Thrombosis and Myocardial Infarct Expansion in Mice. Blood Adv..

[35] Manne B.K., Münzer P., Badolia R., Walker-Allgaier B., Campbell R.A., Middleton E., Weyrich A.S., Kunapuli S.P., Borst O., Rondina M.T. (2018). PDK1 Governs Thromboxane Generation and Thrombosis in Platelets by Regulating Activation of Raf1 in the MAPK Pathway. J. Thromb. Haemost..

[36] Deng Y., Larrivée B., Zhuang Z.W., Atri D., Moraes F., Prahst C., Eichmann A., Simons M. (2013). Endothelial RAF1/ERK Activation Regulates Arterial Morphogenesis. Blood.

[37] Lima A.M., Wegner S.V., Martins Cavaco A.C., Estevão-Costa M.I., Sanz-Soler R., Niland S., Nosov G., Klingauf J., Spatz J.P., Eble J.A. (2018). The Spatial Molecular Pattern of Integrin Recognition Sites and Their Immobilization to Colloidal Nanobeads Determine A2β1 Integrin-Dependent Platelet Activation. Biomaterials.

[38] Chung C.-L., Chen J.-H., Huang W.-C., Sheu J.-R., Hsia C.-W., Jayakumar T., Hsia C.-H., Chiou K.-R., Hou S.-M. (2022). Glabridin, a Bioactive Flavonoid from Licorice, Effectively Inhibits Platelet Activation in Humans and Mice. Int. J. Mol. Sci..

[39] Huang Y.-T., Chen Q.-R., Pan W.-J., Zhang Y., Li J.-S., Xue X.-Y., Lei X.-H., Wang S.-M., Meng J. (2024). Moutan cortex exerts blood-activating and anti-inflammatory effects by regulating coagulation-inflammation cascades pathway in cells, rats and zebrafish. J. Ethnopharmacol..

[40] Woulfe D., Jiang H., Morgans A., Monks R., Birnbaum M., Brass L.F. (2004). Defects in Secretion, Aggregation, and Thrombus Formation in Platelets from Mice Lacking Akt2. J. Clin. Investig..

[41] Yin H., Stojanovic A., Hay N., Du X. (2007). The Roles of Akt1 and Akt2 in GPIb-IX-Mediated Platelet Activation Signaling. Blood.

[42] Ye S., Mao B., Yang L., Fu W., Hou J. (2016). Thrombosis Recanalization by Paeoniflorin through the Upregulation of Urokinase-Type Plasminogen Activator via the MAPK Signaling Pathway. Mol. Med. Rep..

[43] Giri H., Cai X., Panicker S.R., Biswas I., Rezaie A.R. (2019). Thrombomodulin Regulation of Mitogen-Activated Protein Kinases. Int. J. Mol. Sci..

[44] Liu Y., Wang T., Zhou Q., Xin G., Niu H., Li F., Wang Y., Li S., Dong Y., Zhang K. (2023). Endogenous SIRT6 in platelets negatively regulates platelet activation and thrombosis. Front. Pharmacol..

[45] Harper M.-T., Poole A.-W. (2007). Isoform-Specific Functions of Protein Kinase C: The Platelet Paradigm. Biochem. Soc. Trans..

[46] Kondreddy V., Keshava S., Das K., Magisetty J., Rao L.V.M., Pendurthi U.R. (2022). The Gab2–MALT1 Axis Regulates Thromboinflammation and Deep Vein Thrombosis. Blood.

[47] Chion A., Byrne C., Atiq F., Doherty D., Aguila S., Fazavana J., Lopes P., Karampini E., Amin A., Roger J.S.P. (2024). Aptamer BT200 blocks interaction of K1405-1408 in the VWF-A1 domain with macrophage LRP1. Blood.

[48] Yada N., Zhang Q., Bignotti A., Gralnek S.-H., Sosnovske D., Hogan K., Ye Z., Zheng L., Zheng X.-L. (2024). Targeting neutrophil extracellular trap accumulation under flow in patients with immune-mediated thrombotic thrombocytopenic purpura. Blood Adv..

[49] Duval C., Ariëns R.A.S. (2017). Fibrinogen splice variation and cross-linking: Effects on fibrin structure/function and role of fibrinogen γ′ as thrombomobulin II. Matrix Biol..

[50] Mangin P.H., Gardiner E.E., Ariëns R.A.S., Jandrot-Perrus M. (2023). GPVI Interplay with Fibrin(Ogen) in Thrombosis. J. Thromb. Haemost..

[51] Manz X.D., Bogaard H.J., Aman J. (2022). Regulation of VWF (Von Willebrand Factor) in Inflammatory Thrombosis. Arterioscl. Throm. Vas..

[52] Sheng J., Meng Q., Yang Z., Guan J., Zhao Y., Zhang J., Wang Y., Zhao L., Wang Y. (2020). Identification of Cryptotanshinone from Tongmai to Inhibit Thrombosis in Zebrafish via Regulating Oxidative Stress and Coagulation Cascade. Phytomedicine.

[53] Youn K., Ho C.-T., Jun M. (2023). Investigating the Potential Anti-Alzheimer’s Disease Mechanism of Marine Polyphenols: Insights from Network Pharmacology and Molecular Docking. Mar. Drugs.

[54] Li J., Liu H., Yang Z., Yu Q., Zhao L., Wang Y. (2021). Synergistic Effects of Cryptotanshinone and Senkyunolide I in Guanxinning Tablet Against Endogenous Thrombus Formation in Zebrafish. Front. Pharmacol..

[55] Xin S., Zhang M., Li P., Wang L., Zhang X., Zhang S., Mu Z., Lin H., Li X., Liu K. (2024). Marine-Fungus-Derived Natural Compound 4-Hydroxyphenylacetic Acid Induces Autophagy to Exert Antithrombotic Effects in Zebrafish. Mar. Drugs.

