# Supplementary material for: Brevianamide F Exerts Antithrombotic Effects by Modulating the MAPK Signaling Pathway and Coagulation Cascade
Source: Mar Drugs. 2024 Sep 26;22(10):439. doi: 10.3390/md22100439 (PMC11509512; doi:10.3390/md22100439)
Supplement: Supplementary file 1 [file marinedrugs-22-00439-s001.zip › marinedrugs-3210974-supplementary.pdf]

Supplementary Materials

Table S1. The gene primers for Quantitative Real-time PCR.

Gene	Primer orientation	Nucleotide sequence
rpl13a (reference gene)	Forward	5′-TCTGGAGGACTGTAAGAGGTATGC-3′
	Reverse	5′-AGACGCACAATCTTGAGAGCAG-3′
mapk14	Forward	5′-ATCAAACGAGCCTCTGCCAA-3′
	Reverse	5′-CTGCGTAGTCCACCGACTTT-3′
mapk1	Forward	5′-TGAGGCTCCGTTCAAGTTCG-3′
	Reverse	5′-AATCCAGACCAACGCCTGTG-3′
mapk8	Forward	5′-TCCTGGCACAGACCATATTGA-3′
	Reverse	5′-GAGGCCGGTTCTCCACATAC-3′
raf1	Forward	5′-AAGCGCCTTCGAGTAACACT-3′
	Reverse	5′TGCCTGGTCTTCCTGAGAAC-3′
map2k7	Forward	5′-GCAAACGGATCGACCTGAAC-3′
	Reverse	5′-GCTGGATCACGATGATTGGC-3′
mapkapk3	Forward	5′-ACCAAGCTTTCACAGAGCGA-3′
	Reverse	5′-CCTAAAACCTCCGGGGCTAC-3′
mapkapk5	Forward	5′-AGGGTGATTTGATGACCCCG-3′
	Reverse	5′-ACCACAGGTCACAGCTCTTG-3′
mapk11	Forward	5′-CCGGGATTCTACCGGCAAG-3′
	Reverse	5′-CGTCCAGCAGTCCAATAACATTC-3′
akt2	Forward	5′-CACAAAGTCCCGCACCAAAG-3′
	Reverse	5′-GTGCAACTTCGTCCTTAGCG-3′

map3k2	Forward	5′-CAACAAACTGGCGTCTTGGG-3′
	Reverse	5′-CCAGAGCACTCACCTCCTTG-3′
pkcα	Forward	5′-TCGTTGCTTTGTGTATCAGCCATTG-3′
	Reverse	5′-ACCCCCTGATGAAGAGAAGAGAGAA-3′
pkcβ	Forward	5′-CGGCAGAAATTTGAGAGGGC-3′
	Reverse	5′-TTCATCCGGTCTCTGTTGCC-3′
vwf	Forward	5′-GACGCTGTTTCAGTGGTGTC-3′
	Reverse	5′-CATTCGTGAGCATAAGCGGC-3′
f2	Forward	5′-TGCCAAAATGTGGTGAGGCT-3′
	Reverse	5′-GCTTATCACATGACGCTGCTC-3′
f7	Forward	5′-ATCAGGAGCAAACGAGCCAA-3′
	Reverse	5′-CCAGAACTCATTCGTGGCCT-3′
fga	Forward	5′-GGCTTTGTTGGCGGAGATTG-3′
	Reverse	5′-TTGAACATCCCGCTCTGACC-3′
fgb	Forward	5′-AGAAAGTCAGCGAGGGCAAT-3′
	Reverse	5′-ATGTTCTGGGGGAAGGTGAC-3′
fgg	Forward	5′-TCGATCATGCATGTGGTTGC-3′
	Reverse	5′-AGTAGTCTCCTCTTTGCGCTG-3′
